# Practical Points

**Published:** 1895-11-09

**Authors:** 


### PRACTICAL POINTS.

*Disinfector.—Would you kindly inform me where I can get particulars in England of the "Equifex" Disinfector mentioned in your last number, and oblige?—X.*

Apply to A. B. Reck, 155, Gothersgade, Copenhagen, who will send you a statement in English.

